# Dietary behaviors throughout childhood are associated with adiposity and estimated insulin resistance in early adolescence: a longitudinal study

**DOI:** 10.1186/s12966-018-0759-0

**Published:** 2018-12-17

**Authors:** Véronique Gingras, Sheryl L. Rifas-Shiman, Elsie M. Taveras, Emily Oken, Marie-France Hivert

**Affiliations:** 1 Division of Chronic Disease Research Across the Lifecourse, Department of Population Medicine, Harvard Medical School and Harvard Pilgrim Health Care Institute, Landmark Center, 401 Park Drive Suite 401, Boston, MA 02215 USA; 2 Division of General Academic Pediatrics, Department of Pediatrics, Massachusetts General Hospital for Children, Boston, MA USA; 3 Department of Nutrition, Harvard T.H. Chan School of Public Health, Boston, MA USA; 4 Diabetes Unit, Massachusetts General Hospital, Boston, MA USA

**Keywords:** Dietary behaviors, Childhood, Adolescence, Breakfast, Family dinner, Television, Fast-food, Adiposity, Insulin resistance

## Abstract

**Background:**

Despite the growing prevalence of excess weight and prediabetes in children, the contributing role of dietary behaviors throughout childhood remains poorly understood. We examined longitudinal associations of dietary behaviors throughout childhood with adiposity and estimated insulin resistance (HOMA-IR) in adolescence.

**Methods:**

Among 995 children from Project Viva, a pre-birth cohort, we examined associations of child dietary behaviors (frequency of eating breakfast, fast food, family dinner, and eating meals while watching television) reported annually throughout childhood (from ages 4 to 11 years) with body mass index z-score (BMI-z; *n* = 991), waist circumference (WC; *n* = 995), DXA overall and central adiposity measurements (*n* = 721), and HOMA-IR (*n* = 579) in early adolescence (13.2 ± 0.9 years old). We used mixed effects models adjusted for potential confounders.

**Results:**

Eating breakfast daily throughout childhood was associated with lower BMI-z and DXA-measured overall and central adiposity in boys and girls (e.g. for whole-body fat %: β − 1.43% [95% CI: -2.42, − 0.45] and − 1.47% [− 2.25, − 0.68]), and with lower HOMA-IR in boys (% difference − 15.6% [− 22.7, − 7.9]). Daily family dinner and eating fast food less than once per week throughout childhood were both associated with lower BMI-z and adiposity in girls (for BMI-z: β − 0.17 units [− 0.24, − 0.11] and β − 0.09 units [− 0.17, − 0.02]) and lower insulin resistance in boys (% difference − 7.3% [− 12.4,− 1.8] and − 7.6% [− 13.2, − 1.7]). Finally, eating meals while watching television < 1/week throughout childhood was associated with lower adolescent adiposity (e.g. WC: − 1.55 cm [− 2.39, − 0.71]) and HOMA-IR (% difference: − 10.7% [− 15.8, − 5.2]) in boys.

**Conclusion:**

Healthful dietary behaviors throughout childhood are associated with less adiposity and lower estimated insulin resistance in early adolescence.

**Trial registration:**

NCT02820402

**Electronic supplementary material:**

The online version of this article (10.1186/s12966-018-0759-0) contains supplementary material, which is available to authorized users.

## Background

Over the past decades, childhood obesity has become a global epidemic [1], and it is associated with an early appearance of complications that have been traditionally recognized as adult diseases. For example, type 2 diabetes has been increasingly reported in children and adolescents [2, 3] and this increasing prevalence of type 2 diabetes in children parallels the increasing prevalence of obesity and overweight. Pediatric obesity is multifactorial and can be caused by a vast array of genetic, behavioral and environmental factors [4, 5].

Several lifestyle components including unhealthy dietary behaviors have been associated with the rising prevalence of childhood obesity and related cardiometabolic disorders [6]. However, previous observational reports often did not account for potential confounders and did not have longitudinal assessment over multiple time points during childhood to investigate long-term effects of lifestyle behaviors. In addition, little is known about the contributing role of dietary behaviors to insulin resistance and type 2 diabetes in children, and whether these associations, if present, are independent of adiposity.

The aim of this study was to examine longitudinal associations of dietary behaviors including frequency of eating breakfast, family dinner, eating fast food, and eating meals while watching television, throughout childhood with obesity, body composition, and estimated insulin resistance in early adolescence. A better understanding of the long-term effects of these dietary behaviors on risk of childhood obesity and insulin resistance could help improve the focus of family-based interventions in children.

## Methods

### Subjects

We studied participants from Project Viva, a longitudinal pre-birth cohort from eastern Massachusetts, United States, established to examine associations of pre- and postnatal factors with maternal and child health. Between 1999 and 2003, we recruited women who understood English, were at < 22 weeks of pregnancy and with a singleton pregnancy during their first prenatal visit at one of the participating clinics from Atrius Harvard Vanguard Medical Associates [7]. We previously published a full cohort description, including recruitment methods [7] and we registered Project Viva as NCT02820402. All study instruments and protocols are available online, at https://www.hms.harvard.edu/viva/protocol-policies.html.

Women who agreed to participate completed in-person visits during early pregnancy, mid-pregnancy, and at delivery. Mothers and children then completed in-person visits in infancy (6 months), early (mean age, 3.2 years old), and mid-childhood (mean age, 7.9 years old) as well as early adolescence (mean age, 13.2 years). Every year beginning at 1 year postpartum, we mailed questionnaires to mothers, and we sent questionnaires to children as well starting at age nine years. From the original 2128 live-born singletons, 1684 remained eligible (did not disenroll) for the early teen visit and 1038 completed the in-person study visit. Of 1038, we excluded 7 with missing covariate data and 36 with no data for dietary behaviors throughout childhood, thus the final analysis sample included 995 participants (Additional file 1: Figure S1).

The 995 included compared to the 1133 excluded participants from the initial cohort were similar in terms of maternal pre-pregnancy BMI (mean 24.8 vs. 25.0 kg/m^2^), marital status (92 vs. 91% married or living together), parity (47% vs. 48% nulliparous), and child race/ethnicity (65 vs. 63% white). Women included were more likely to have completed a college degree (71 vs. 59%) and had higher annual household income (64 vs. 58% reported > US $70,000/year).

The institutional review board of Harvard Pilgrim Health Care approved this study and all procedures were in accordance with the ethical standards for human experimentation established by the Declaration of Helsinki. All mothers provided written informed consent at enrollment and at each postpartum in-person visit, and children provided verbal assent at the early teen visit.

### Measurements

#### Exposures

We obtained data on dietary behaviors from self-administered questionnaires. We sent yearly questionnaires (by mail and/or email) to mothers who were still enrolled in the cohort, except for at the mid-childhood when the questionnaires were self-administered during in-person visits at home or at the research facility. Starting at age nine years, participating children themselves also completed yearly questionnaires. Thus, for this study, the exposures are based on maternal reports from ages four to eight years and child reports from ages nine to eleven years. Although questionnaires were sent starting at one-year postpartum, the dietary behaviors of interest in this analysis (exposures) were included in the questionnaires beginning at age four. The number of collected questionnaires at each visit from age four to age eleven ranged from 584 to 916 (59 to 92% of included sample per year; Additional file 1: Figure S2).

We examined four dietary behaviors in this study: eating breakfast, eating dinner together with family, eating fast food, and eating meals while watching television. We questioned participants on the average frequency of these behaviors over the previous month. For example, for family dinner, the question was: *In the past month, on average, how often does your child eat supper or dinner together with family members (“family dinner”)?* Answers ranged on a five-level scale, from “never/less than once per week” to either “every day” or “five times per week or more”, depending on the question. For analysis purposes, we categorized eating breakfast and eating dinner together with family as “always/daily” versus “≤ six times per week”. We categorized fast food consumption and eating meals while watching television as “less than once per week (between zero and three times per month)” versus “≥ once per week”. Categorization was based upon available choices of answers in questionnaires through time, and so that for each behavior we were comparing the hypothesized beneficial direction of exposure compared with the less beneficial exposure. All four behaviors were reported annually from age four to eleven except for breakfast eating, which we did not assess at age eleven.

#### Outcomes

Outcomes included children’s adiposity measurements and estimated insulin resistance at early adolescence (mean ± SD: 13.2 ± 0.9 years old). Research assistants performing all measurements completed regular training and used standardized techniques. During the in-person visit, we measured weight (TBF-300A scale, Tanita, Arlington Heights, IL) and standing height (calibrated stadiometer, Shorr Productions, Olney, MD). We calculated age and sex-specific BMI z-scores (BMI-z) using U.S. national reference data [8]. We measured waist circumference just above the iliac crest in standing participants with waist exposed using a Gulick II measuring tape (Performance Health, Warrenville, IL) [9]. We measured whole-body fat percentage with bio-impedance (BI) analysis using the TBF-300A scale. We also performed dual-energy X-ray absorptiometry (DXA) scans (Hologic model Discovery A, Bedford, MA) for participants who completed the in-person visit at the research facility (*n* = 721). A quality control check of the DXA scan (inspection and daily calibration with a standard phantom from the manufacturer to assess for machine drift), as per manufacturer’s instructions, was completed on every day with in-person visits and Hologic software version 4.0 was used for scan analysis. The same DXA scanner was used on all participants, and a single trained research assistant verified all scans and defined body regions for analysis. Whole-body fat percentage, trunk fat mass and trunk to peripheral fat mass ratio were derived from the DXA scan analysis [10]. A phlebotomist performed fasting blood draw in willing participants at early adolescence (*n* = 579). We requested that children were fasting for at least eight hours before the time of the blood draw. Plasma fasting insulin was measured using an electro-chemiluminescence immunoassay on the Roche Modular system and fasting glucose was measured enzymatically using Roche Diagnostics reagents. We estimated insulin resistance using the homeostatic model assessment (HOMA-IR; (glucose in mg/dL x insulin in μU/mL) / 405) [11].

#### Covariates

At enrollment during the first trimester of pregnancy (median 9.9 weeks), women reported their age, education level, parity, marital status, household income, height, and pre-pregnancy weight, from which we calculated pre-pregnancy BMI (kg/m^2^). We obtained child’s sex from the delivery interview and mothers reported their child’s race/ethnicity at the early childhood (3-year) visit.

### Statistical analysis

**Fig. 1:**
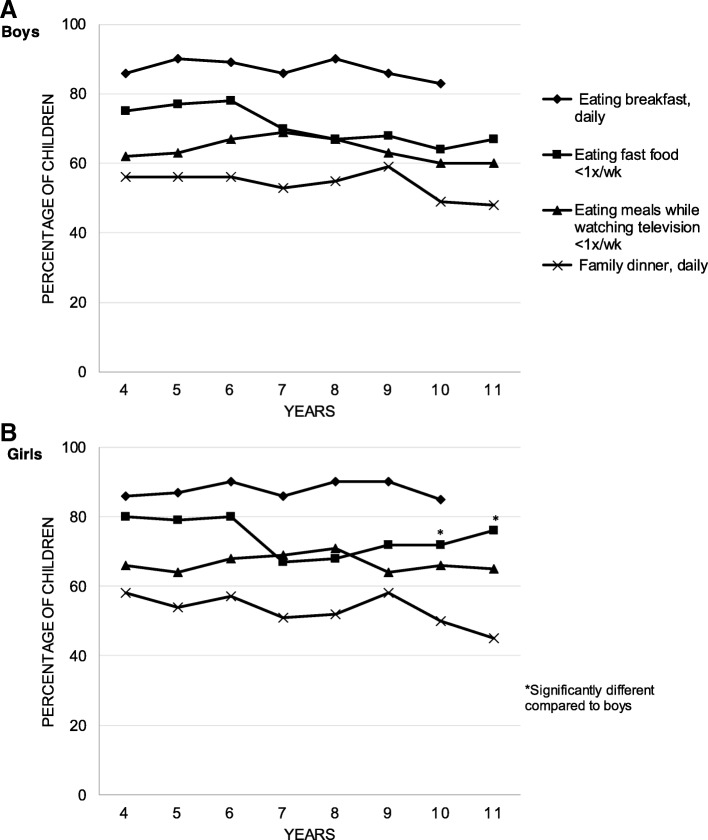
Trends in dietary behaviors in boys (**a**) and girls (**b**) throughout childhood (*N* = 297 to 461 for boys and *N* = 284 to 455 for girls)

We categorized characteristics of mothers and children as presented in Table 1 and presented descriptive characteristics for mothers at inclusion and children in early adolescence as mean ± SD or number (%). We calculated annual prevalence of each of the four dietary behaviors of interest including all children with at least one early adolescence outcome, and present sex-specific prevalences from ages 4 to 11 (4 to 10 for daily breakfast) in Fig. 1. We examined whether the proportions of participants adopting each behavior varied yearly using McNemar’s tests. We examined associations of maternal and child characteristics at inclusion with dietary behaviors throughout childhood (age 4 to 11) separately in boys and girls using general linear mixed models (PROC GLIMMIX) with a logistic link function (Table 2). We estimated the longitudinal associations of childhood dietary behaviors from age 4 to 11 with adiposity measurements and HOMA-IR in early adolescence using multivariate linear mixed effect models, with the children’s age at exposure and covariates as fixed effects, and subject as a random effect (Table 3). The first model was adjusted for age at outcome, while model 2 was additionally adjusted for baseline maternal socio-demographic characteristics as well as child’s race/ethnicity. Finally, model 3 was additionally adjusted for maternal pre-pregnancy BMI. We assessed sex interactions in the longitudinal models and we found significant interactions across all behaviors. As a result, we conducted all analyses stratified by sex. Because HOMA-IR was right skewed, we log-transformed this outcome prior to including in the linear mixed model. We also presented associations with HOMA-IR additionally adjusted for current BMI at early adolescence. To simplify the interpretation, we exponentiated regression coefficients and confidence intervals and we presented them as percent change. We conducted all analyses using SAS version 9.4 (SAS Institute).

**Table 1:** Maternal baseline characteristics and children’s characteristics in early adolescence (*N* = 995)

Maternal characteristics	Overall (*N* = 995)	Boys (*N* = 504)	Girls (*N* = 491)
Age, years	32.4 ± 5.1	32.2 ± 5.3	32.6 ± 4.8
Annual household income, US$^a^			
> 70,000	589 (64)	302 (66)	287 (63)
≤ 70,000	327 (36)	155 (34)	172 (37)
Maternal education			
≥ College degree	709 (71)	348 (69)	361 (74)
< College degree	286 (29)	156 (31)	130 (26)
Mother’s marital status			
Married / cohabitating	916 (92)	466 (92)	450 (92)
Other	79 (8)	38 (8)	41 (8)
Parity			
Nulliparous	472 (47)	239 (47)	233 (47)
≥ 1 child	523 (53)	265 (53)	258 (53)
Pre-pregnancy BMI, kg/m^2^	24.8 ± 5.2	24.8 ± 4.9	24.7 ± 5.5
Pre-pregnancy BMI			
≥ 25 kg/m^2^	367 (37)	197 (39)	170 (35)
< 25 kg/m^2^	628 (63)	307 (61)	321 (65)
Child characteristics	Overall	Boys	Girls
Race / ethnicity			
White	647 (65)	315 (63)	332 (68)
Black	29 (3)	16 (3)	13 (3)
Asian	157 (16)	85 (17)	72 (15)
Hispanic	45 (5)	24 (5)	21 (4)
Other	117 (12)	64 (13)	53 (11)
Age at early adolescence visit, years	13.2 ± 0.9	13.2 ± 1.0	13.2 ± 0.9
BMI Z-score, unit^b^	0.37 ± 1.06	0.39 ± 1.06	0.34 ± 1.06
Waist circumference, cm	73.0 ± 11.6	73.3 ± 12.1	72.8 ± 11.1
BI whole-body fat, %^c^	21.8 ± 10.3	17.5 ± 9.3	26.2 ± 9.4
DXA whole-body fat, %^d^	28.6 ± 7.5	26.8 ± 8.0	30.4 ± 6.6
DXA trunk fat, kg^d^	6.2 ± 4.0	5.7 ± 4.0	6.6 ± 3.9
Trunk to peripheral fat ratio^d^	0.59 ± 0.12	0.57 ± 0.11	0.61 ± 0.12
Fasting glucose, mg/dl^e^	92.9 ± 23.5	93.2 ± 18.3	92.6 ± 28.1
Fasting insulin, uU/ml^e^	14.1 ± 9.0	12.7 ± 9.0	15.7 ± 8.9
HOMA-IR^e^	3.23 ± 2.43	2.88 ± 2.18	3.62 ± 2.63

Presented as mean ± SD or N (%); *BMI* Body mass index, *BI* Bio-impedance, *DXA* Dual-energy X-ray absorptiometry, *HOMA-IR* Homeostatic model assessment for insulin resistance

^a^*N* = 916;^b^ *N* = 991;^c^ *N* = 982;^d^ *N* = 721;^e^ *N* = 579

**Table 2:** Associations of maternal and child characteristics with dietary behaviors among Viva children throughout childhood (ages 4 to 11 years; *N* = 995)

	Eating breakfast daily		Eating dinner with family daily		Eating fast food < 1/wk		Eating meals while watching television < 1/wk	
	OR (95% CI)							
	Boys	Girls	Boys	Girls	Boys	Girls	Boys	Girls
Maternal characteristics								
Household income								
> 70,000 US$	1.0 (ref)	1.0 (ref)	1.0 (ref)	1.0 (ref)	1.0 (ref)	1.0 (ref)	1.0 (ref)	1.0 (ref)
≤ 70,000 US$	0.55 (0.39, 0.76)	0.56 (0.40, 0.79)	0.97 (0.77, 1.22)	1.04 (0.83, 1.31)	0.87 (0.68, 1.11)	0.58 (0.45, 0.73)	0.76 (0.60, 0.98)	0.61 (0.48, 0.78)
Maternal education								
≥ College degree	1.0 (ref)	1.0 (ref)	1.0 (ref)	1.0 (ref)	1.0 (ref)	1.0 (ref)	1.0 (ref)	1.0 (ref)
< College degree	0.43 (0.31, 0.59)	0.45 (0.32, 0.63)	0.91 (0.73, 1.15)	0.76 (0.59, 0.97)	0.61 (0.48, 0.77)	0.49 (0.38, 0.64)	0.54 (0.42, 0.69)	0.46 (0.35, 0.60)
Maternal marital status								
Married / cohabitating	1.0 (ref)	1.0 (ref)	1.0 (ref)	1.0 (ref)	1.0 (ref)	1.0 (ref)	1.0 (ref)	1.0 (ref)
Other	0.35 (0.21, 0.59)	0.39 (0.24, 0.64)	1.03 (0.66, 1.61)	0.64 (0.42, 0.98)	0.91 (0.57, 1.46)	0.56 (0.37, 0.85)	0.52 (0.33, 0.83)	0.45 (0.29, 0.68)
Maternal parity								
≥ 1	1.0 (ref)	1.0 (ref)	1.0 (ref)	1.0 (ref)	1.0 (ref)	1.0 (ref)	1.0 (ref)	1.0 (ref)
Nulliparous	1.03 (0.76, 1.41)	1.37 (0.99, 1.90)	1.02 (0.83, 1.26)	1.15 (0.93, 1.43)	1.14 (0.91, 1.42)	1.24 (0.98, 1.56)	0.86 (0.68, 1.07)	1.02 (0.81, 1.28)
Maternal pre-pregnancy BMI								
< 25 kg/m^2^	1.0 (ref)	1.0 (ref)	1.0 (ref)	1.0 (ref)	1.0 (ref)	1.0 (ref)	1.0 (ref)	1.0 (ref)
≥ 25 kg/m^2^	0.72 (0.53, 0.99)	0.65 (0.46, 0.90)	1.02 (0.82, 1.26)	0.77 (0.61, 0.96)	0.81 (0.64, 1.02)	0.57 (0.45, 0.73)	0.79 (0.63, 0.99)	0.69 (0.54, 0.88)
Child characteristics								
Race/ethnicity								
White	1.0 (ref)	1.0 (ref)	1.0 (ref)	1.0 (ref)	1.0 (ref)	1.0 (ref)	1.0 (ref)	1.0 (ref)
Asian	0.64 (0.28, 1.49)	0.42 (0.19, 0.94)	0.84 (0.47, 1.49)	2.63 (1.26, 5.50)	0.71 (0.39, 1.30)	1.53 (0.68, 3.43)	0.89 (0.48, 1.67)	2.04 (0.86, 4.85)
Black	0.38 (0.26, 0.56)	0.35 (0.23, 0.53)	0.72 (0.53, 0.97)	0.68 (0.49, 0.95)	0.50 (0.37, 0.68)	0.55 (0.39, 0.77)	0.52 (0.38, 0.71)	0.38 (0.27, 0.53)
Hispanic	0.48 (0.24, 0.94)	0.28 (0.14, 0.55)	1.34 (0.79, 2.27)	0.99 (0.54, 1.80)	0.90 (0.51, 1.59)	0.30 (0.17, 0.53)	0.95 (0.54, 1.66)	0.43 (0.23, 0.79)
Other	0.48 (0.31, 0.75)	1.04 (0.58, 1.85)	1.04 (0.76, 1.43)	1.04 (0.73, 1.47)	0.94 (0.66, 1.34)	0.80 (0.55, 1.15)	0.92 (0.65, 1.30)	0.49 (0.34, 0.71)

**Table 3:** Longitudinal associations of dietary behaviors throughout childhood (ages 4 to 11 years) with adiposity and estimated insulin resistance in early adolescence (*N* = 995)

	Boys			Girls		
	β (95% CI)			β (95% CI)		
	Model 1^a^	Model 2^b^	Model 3^c^	Model 1^a^	Model 2^b^	Model 3^c^
BMI, Z-Score	*N* = 502			*N* = 489		
Eating breakfast daily	−0.22 (− 0.34, − 0.11)	− 0.18 (− 0.30, − 0.06)	− 0.13 (− 0.24, − 0.02)	−0.36 (− 0.48, − 0.24)	− 0.19 (− 0.31, − 0.07)	− 0.13 (− 0.23, − 0.02)
Family dinner daily	− 0.03 (− 0.11, 0.04)	−0.01 (− 0.09, 0.06)	−0.01 (− 0.09, 0.06)	−0.26 (− 0.33, − 0.18)	−0.21 (− 0.29, − 0.14)	−0.17 (− 0.24, − 0.11)
Fast food restaurant < 1/wk	− 0.11 (− 0.19, − 0.03)	−0.07 (− 0.15, 0.02)	−0.04 (− 0.12, 0.04)	−0.29 (− 0.38, − 0.21)	−0.19 (− 0.27, − 0.10)	−0.09 (− 0.17, − 0.02)
Eating meals while watching television < 1/wk	− 0.21 (− 0.29, − 0.13)	− 0.16 (− 0.24, − 0.08)	− 0.13 (− 0.20, − 0.05)	−0.21 (− 0.29, − 0.13)	−0.06 (− 0.14, 0.01)	−0.03 (− 0.10, 0.04)
Waist circumference, cm	*N* = 504			*N* = 491		
Eating breakfast daily	−1.29 (− 2.62, 0.03)	− 1.11 (− 2.44, 0.23)	− 0.60 (− 1.87, 0.68)	−3.98 (−5.21, − 2.74)	− 2.29 (− 3.48, − 1.10)	− 1.59 (− 2.67, − 0.51)
Family dinner daily	0.06 (− 0.78, 0.91)	0.14 (− 0.69, 0.98)	0.13 (− 0.67, 0.93)	− 2.12 (− 2.89, − 1.36)	−1.57 (− 2.29, − 0.84)	−1.14 (− 1.80, − 0.48)
Fast food restaurant < 1/wk	− 0.87 (− 1.80, 0.06)	− 0.71 (− 1.64, 0.22)	− 0.33 (− 1.22, 0.56)	− 3.21 (− 4.07, − 2.34)	− 2.17 (− 3.00, − 1.35)	−1.23 (− 1.99, − 0.48)
Eating meals while watching television < 1/wk	− 2.14 (− 3.01, − 1.27)	−1.94 (− 2.82, − 1.07)	−1.55 (− 2.39, − 0.71)	−2.44 (− 3.25, − 1.64)	− 1.04 (− 1.81, − 0.26)	− 0.70 (− 1.40, 0.01)
BI whole-body fat, %	*N* = 499			*N* = 483		
Eating breakfast daily	−1.94 (− 2.98, − 0.90)	−1.38 (− 2.42, − 0.34)	− 0.94 (− 1.92, 0.05)	−3.63 (− 4.69, − 2.57)	−1.98 (− 2.98, − 0.97)	−1.47 (− 2.39, − 0.54)
Family dinner daily	− 0.08 (− 0.75, 0.58)	0.11 (− 0.54, 0.77)	0.09 (− 0.53, 0.71)	− 2.17 (− 2.83, − 1.51)	−1.66 (− 2.28, − 1.04)	−1.34 (− 1.91, − 0.77)
Fast food restaurant < 1/wk	− 0.91 (− 1.64, − 0.18)	− 0.46 (− 1.19, 0.27)	−0.14 (− 0.82, 0.55)	−2.43 (− 3.17, − 1.68)	−1.39 (− 2.09, − 0.69)	− 0.64 (− 1.29, 0.01)
Eating meals while watching television < 1/wk	− 2.09 (− 2.77, − 1.41)	−1.66 (− 2.35, − 0.98)	−1.33 (− 1.98, − 0.69)	−2.32 (− 3.01, − 1.62)	−0.98 (− 1.64, − 0.32)	−0.74 (− 1.35, − 0.14)
DXA whole-body fat, %	*N* = 355			*N* = 366		
Eating breakfast daily	−1.78 (− 2.80, − 0.76)	−1.75 (− 2.78, − 0.72)	−1.43 (− 2.42, − 0.45)	−2.34 (− 3.21, − 1.47)	− 1.93 (− 2.77, − 1.09)	−1.47 (− 2.25, − 0.68)
Family dinner daily	−0.10 (− 0.77, 0.57)	−0.04 (− 0.71, 0.63)	−0.16 (− 0.80, 0.48)	−0.59 (− 1.14, − 0.04)	−0.49 (− 1.02, 0.03)	−0.29 (− 0.78, 0.19)
Fast food restaurant < 1/wk	− 0.07 (− 0.79, 0.65)	−0.01 (− 0.73, 0.72)	0.21 (− 0.48, 0.90)	−1.78 (− 2.40, − 1.15)	−1.28 (− 1.88, − 0.68)	−0.89 (− 1.45, − 0.33)
Eating meals while watching television < 1/wk	− 1.30 (− 1.99, − 0.62)	−1.25 (− 1.94, − 0.55)	−1.10 (− 1.77, − 0.44)	−0.88 (− 1.46, − 0.30)	−0.39 (− 0.95, 0.17)	−0.15 (− 0.67, 0.37)
DXA trunk fat mass, kg	*N* = 355			*N* = 366		
Eating breakfast daily	−0.65 (− 1.16, − 0.15)	−0.57 (− 1.08, − 0.06)	−0.40 (− 0.88, 0.08)	−1.78 (− 2.26, − 1.29)	−1.23 (− 1.69, − 0.78)	− 0.92 (− 1.33, − 0.51)
Family dinner daily	0.13 (−0.20, 0.46)	0.16 (− 0.16, 0.49)	0.10 (− 0.21, 0.41)	−0.60 (− 0.91, − 0.30)	−0.45 (− 0.74, − 0.16)	−0.32 (− 0.57, − 0.06)
Fast food restaurant < 1/wk	−0.10 (− 0.45, 0.26)	−0.01 (− 0.36, 0.35)	0.11 (− 0.22, 0.44)	− 1.29 (− 1.64, − 0.94)	−0.86 (− 1.19, − 0.53)	−0.60 (− 0.90, − 0.31)
Eating meals while watching television < 1/wk	−0.71 (− 1.05, − 0.38)	−0.63 (− 0.97, − 0.29)	−0.56 (− 0.88, − 0.23)	−0.74 (− 1.06, − 0.42)	−0.31 (− 0.61, 0.00)	−0.15 (− 0.42, 0.12)
Trunk to peripheral fat ratio	*N* = 355			*N* = 366		
Eating breakfast daily	−0.02 (− 0.04, − 0.01)	−0.02 (− 0.04, − 0.01)	−0.02 (− 0.03, − 0.01)	−0.06 (− 0.08, − 0.05)	−0.06 (− 0.07, − 0.04)	−0.05 (− 0.06, − 0.03)
Family dinner daily	0.01 (0.00, 0.02)	0.01 (0.00, 0.02)	0.01 (0.00, 0.02)	−0.02 (− 0.03, − 0.01)	−0.02 (− 0.03, − 0.01)	−0.02 (− 0.03, − 0.01)
Fast food restaurant < 1/wk	−0.01 (− 0.02, 0.00)	−0.01 (− 0.02, 0.00)	0.00 (− 0.01, 0.00)	−0.04 (− 0.05, − 0.03)	−0.03 (− 0.04, − 0.02)	−0.03 (− 0.04, − 0.02)
Eating meals while watching television < 1/wk	−0.02 (− 0.03, − 0.01)	−0.02 (− 0.03, − 0.01)	−0.02 (− 0.03, − 0.01)	−0.03 (− 0.04, − 0.02)	−0.02 (− 0.03, − 0.01)	−0.01 (− 0.02, 0.00)
	% difference (95% CI)			% difference (95% CI)		
HOMA-IR	*N* = 304			*N* = 275		
Eating breakfast daily	−20.1 (− 26.8, − 12.9)	−17.7 (− 24.7, − 10.1)	− 15.6 (− 22.7, − 7.9)	− 17.1 (− 23.8, − 9.7)	−9.2 (− 16.5, − 1.2)	−7.8 (− 15.1, 0.1)
Family dinner daily	−8.0 (− 13.2, − 2.6)	−6.8 (− 12.0, − 1.3)	−7.3 (− 12.4, − 1.8)	−7.0 (− 12.0, − 1.7)	− 3.1 (− 8.2, 2.2)	− 3.2 (− 8.1, 2.1)
Fast food restaurant < 1/wk	− 10.1 (− 15.5, − 4.3)	−8.3 (− 13.9, − 2.4)	− 7.6 (− 13.2, − 1.7)	−13.3 (− 18.5, − 7.7)	−8.5 (− 13.8, − 2.8)	−6.6 (− 12.0, − 0.9)
Eating meals while watching television < 1/wk	− 13.9 (− 18.9, − 8.6)	− 11.7 (− 16.8, − 6.2)	−10.7 (− 15.8, − 5.2)	−3.4 (− 8.8, 2.3)	3.1 (−2.5, 9.1)	3.7 (− 1.8, 9.6)

*BMI* Body mass index, *HOMA-IR* Homeostatic model assessment for insulin resistance, *BI* Bio-impedance, *DXA* Dual-energy X-ray absorptiometry. ^a^Adjusted for child’s age at exposures and outcome (except for BMI Z-Score); ^b^Model 1 additionally adjusted for child race/ethnicity and maternal education, marital status, and parity, ^c^Model 2 additionally adjusted for pre-pregnancy BMI

## Results

Our sample included 504 boys and 491 girls for whom at least one outcome was measured during early adolescence (mean ± SD: 13.2 ± 0.9 years old). Children were mostly white (65%) and the majority of mothers had a high education level (71% ≥ college degree) and household income (64% > 70,000 $US), and 92% were married or cohabitating (Table 1).

The frequency of healthful dietary behaviors either remained stable or decreased slightly with age in both sexes, although not in a linear fashion. Daily breakfast intake remained quite stable from age 4 (86%) to age 10 (84%; *P* = 0.12). Daily family dinner decreased, from 57% at age 4 to 46% at age 11 (*P* < 0.001); its decrease mainly started around 9 years old. Fast food consumption less than once per week, decreased slightly, from 77 to 71% (*P* < 0.001), with a first drop between 6 and 7 years. Finally, eating meals while watching television less than once per week remained stable, 64 and 63% at ages 4 and 11 (*P* = 0.32). The proportion of boys eating fast food less than once per week was lower than for girls at 10 (64 vs. 72%; *P* = 0.02) and 11 years old (67 vs. 76%; *P* = 0.01). No other sex-specific differences were observed in frequency of healthful dietary behaviors during childhood.

Both child and maternal characteristics were associated with dietary behaviors in both boys and girls (Table 2). Child’s race / ethnicity was associated with all four examined dietary behaviors. A lower household income was associated with lower odds of eating breakfast daily, eating fast food less than once per week and eating meals while watching television less than once per week. A lower maternal education level, not being married or in partnership, and being overweight/obese pre-pregnancy were additionally associated with a lower odds of eating family dinner daily. Parity was not associated with dietary behaviors throughout childhood.

We present sex-stratified adjusted longitudinal associations of dietary behaviors throughout childhood, from age 4 to 11, with early adolescent (age 13.2 ± 0.9 years) adiposity and estimated insulin resistance in Table 3. Overall, the effect estimates for all associations were reduced after adjustment for socio-economic characteristics, and further attenuated after adjustment for maternal pre-pregnancy BMI, although several associations remained significant. In fully adjusted models, eating breakfast daily was associated with lower adiposity measurements (BMI-z, DXA whole-body fat percentage and trunk to peripheral fat ratio) in both boys and girls, as well as with lower HOMA-IR in boys and with lower waist circumference, BI whole-body fat percentage and DXA trunk fat mass in girls. The association with HOMA-IR in boys remained significant after adjustment for current BMI (BMI-adjusted % difference: − 12.9%; 95% CI [− 19.5, − 5.7]). Daily family dinner was associated with lower adiposity (BMI-z, waist circumference, BI whole-body fat percentage, DXA trunk fat mass and trunk to peripheral fat ratio) in girls. In boys, daily family dinner was associated with lower HOMA-IR, an association that remained significant after adjustment for current BMI (BMI-adjusted % difference; − 5.5 95% CI [− 10.2, − 0.5]). Eating fast food less than once per week was associated with lower adiposity (BMI-z, waist circumference and DXA adiposity measurements) in girls as well as with lower HOMA-IR only in boys and girls; however, the associations with HOMA-IR were attenuated after adjustment for current BMI (BMI-adjusted % difference: − 4.5%; 95% CI [− 9.7, 1.0] in boys and − 4.7% [− 9.9, 0.8] in girls). Associations of eating meals while watching television with adiposity were mainly seen in boys: eating meals while watching television less than once per week throughout childhood was associated with lower adiposity (BMI-z, waist circumference, BI whole-body fat percentage, and DXA adiposity measurements) and HOMA-IR in boys, an association that was only slightly attenuated when adjusted for current BMI (BMI-adjusted % difference: − 7.7%; 95% CI [− 12.5, − 2.6]). In girls, eating meals while watching television less than once per week throughout childhood was associated with lower BI whole-body fat percentage.

## Discussion

In this longitudinal cohort, the proportion of children with healthful dietary behaviors decreased slightly from childhood into early adolescence in a non-linear trend, and with only minor differences between girls and boys. Dietary behaviors were associated with child and maternal characteristics as well as socio-economic status. We observed several sex-specific associations between dietary behaviors throughout childhood and adiposity and insulin resistance in early adolescence. Eating breakfast every day was associated with lower adiposity, both overall (BMI-z, whole-body fat percentage) and central (trunk to peripheral fat ratio) in boys and girls, and with lower insulin resistance in boys. Daily family dinner and eating fast food less than once per week were both associated with lower adiposity in girls and lower insulin resistance in boys. Finally, eating meals while watching television was associated with adiposity and insulin resistance mainly in boys.

Our results are mostly in line with previously published observational reports for trends in dietary behaviors during childhood, but add to current literature with our longitudinal assessments. Breakfast frequency in children and adolescents was investigated in several studies in various populations, and these studies consistently showed a decrease in breakfast consumption from childhood to adolescence [12–15]. Important variations in breakfast eating across countries and regions have been reported, and lower odds of eating breakfast daily were observed in girls, children from a family with a lower socio-economic status and a family structure that did not include two parents [12]. Throughout childhood, we found an even proportion of children eating breakfast daily between ages four and ten; however, we found an association of daily breakfast eating with maternal household income, education and marital status, as well as with maternal BMI and child’s race/ethnicity, but not with child’s sex. Observational and longitudinal studies in children have suggested that regular breakfast consumption plays a role in the prevention of excess adiposity and obesity during childhood and adolescence [15], despite higher energy intakes with regular breakfast consumption [13]. The overall nutritional profile is typically improved with regular breakfast eating, including higher intakes of micronutrients, fruits, whole grains and dairy [16]. Eating breakfast could also impact global dietary quality and energy intakes beyond breakfast itself by influencing hunger and satiety for the remaining meals [15]. However, results from intervention studies including breakfast initiation as a weight loss strategy have yielded inconclusive results [17] and a recent review suggested positive to neutral support for the inclusion of breakfast for improvements in appetite control, satiety, and postprandial energy expenditure [18]. In the present study, we showed that daily breakfast consumption throughout childhood had the strongest association of all behaviors examined with lower adiposity in early adolescence in both boys and girls, and we added to the previous studies by demonstrating an association with lower insulin resistance in boys. Our findings thus corroborate results from previous observational studies, and our longitudinal assessment suggest that long-term breakfast consumption, starting at an earlier age could be a key element explaining some of the breakfast benefits. Our results also suggest that amongst childhood dietary behaviors, breakfast habits could possibly be one of the most important for both boys and girls on which to focus interventions.

The benefits of family dinner have also been studied quite extensively. For example, in the Growing Up Today Study (GUTS), 50.7% of children ate dinner together with family every day at age 9, a proportion that decreased to 35.4% by age 14 [19]. Although the proportion of children eating dinner with their family daily was slightly higher in our study sample at age 9 (58%), a similar decrease was observed with 46% of children eating dinner with their family daily by age 11. Eating meals together with family may contribute to healthful dietary intakes such as increased fruit and vegetables consumption and less fried food and soda [19–21]. In addition, family dinner frequency has been associated with childhood obesity in some [19, 21], but not all studies [22]. In GUTS, the frequency of eating dinner together with family was not associated with the likelihood of becoming overweight in longitudinal analyses and no sex-specific differences were reported [22]. In contrast, we found an association between daily family dinner throughout childhood and BMI-z as well as some adiposity measurements in girls in early adolescence. This discrepancy could be explained by the children’s age at assessment. In GUTS, children were 9 to 14 years of age at inclusion while we included children starting at 4 years old, thus suggesting greater effects of this behavior if sustained starting at younger ages.

Fast food consumption has on average increased over the past decades and increases with age across childhood and adolescence [23, 24]. In the 2011–2012 National Health and Nutrition Examination Survey (NHANES), a lower caloric intake from fast food was found in non-Hispanic Asian children and no differences were noted by sex and poverty status [24]. Fast food meals and food items are typically energy-dense and have a poor nutrient profile. Fast food consumption in children has been associated with higher energy, fat, sodium, and sugar intakes as well as lower intakes of fruits, vegetables and fiber [25, 26]. Results from cross-sectional studies in children investigating the association between higher fast food intake and BMI are somewhat inconsistent [27], but results from GUTS (9 to 14 years old) showed that a yearly increase (from never or < once per week to 4–7 times per week) in the consumption of fried food away from home was associated with higher BMI compared to children with a constant low intake of fried food away from home, with similar findings between girls and boys [28]. However, we found associations of fast food consumption throughout childhood with adiposity only in girls and with insulin resistance in boys and girls. Mechanisms including caloric intake compensation or responsiveness to internal cues of hunger and satiety should be investigated to try and explain these differences between boys and girls.

To our knowledge, no study has reported trends in eating meals while watching television across childhood or associations of eating while watching television with adolescence obesity or adiposity. Watching television itself has been associated with obesity, in part due to increased sedentary time, but it could also be related to dietary behaviors and choices [29, 30]. Moreover, eating meals or snacks while watching television is common in children and it has been associated with increased energy intakes, poorer diet quality and increased intakes of sugar and fat, snack food, and sugar-sweetened beverages, as well as lower intakes of fruits and vegetables [31, 32]. These effects of eating while watching television could be explained by the increased exposure to unhealthy food advertisement targeting children [33] or to the effects of “mindless or inattentive eating” [34]. Again, we observed sex-specific associations for this behavior, with eating meals while watching television being associated with adiposity and insulin resistance mainly in boys. Future studies should examine underlying mechanisms explaining these sex-related differences.

A key finding of our study was that several healthful dietary behaviors throughout childhood were associated with lower estimated insulin resistance in boys in early adolescence, some independently of BMI. Type 2 diabetes is a growing health concern in children [35]. Unhealthy lifestyle behaviors during childhood combined with increased body weight likely contribute to the risk of prediabetes; however, the contributing role of dietary behaviors throughout childhood remains scarcely studied. Our results indicate that daily breakfast eating, daily family dinner, and eating meals while watching television less than once per week throughout childhood were associated with lower early adolescence HOMA-IR in boys, even after adjustment for current BMI-z, suggesting potential benefits of these behaviors beyond weight control. Our results support the need for nutrition education for diabetes prevention in youth.

Our findings also highlight the importance of adopting healthful dietary behaviors early on, and maintaining them throughout childhood. Other studies have shown that behaviors set in childhood are likely to become habits and carry into adulthood, further increasing the long-course benefits of early healthful behaviors [36–38]. For example, the longitudinal association between diet quality and lifestyle behaviors was examined in the NEXT Plus Study [36]. Over a 4-year period from adolescence to adulthood, breakfast, family meals and eating meals while watching television decreased while fast food consumption increased. Breakfast and family meals as well as less frequent fast food and meals during television viewing throughout late adolescence were all associated with healthier diet quality in young adulthood [36].

An expert committee including representatives of 15 national health care organizations published recommendations regarding the prevention, assessment and treatment of child and adolescent overweight and obesity [39]. Their recommendations included a list of healthful lifestyle habits recognized as effective in preventing excessive weight gain. The following behaviors were included in this list: eating breakfast daily, limiting eating out at restaurants, particularly fast food restaurants, and encouraging family meals in which parents and children eat together [39]. Avoiding eating while watching television was not included in the list, although limiting screen and television time was. Our results provide empiric evidence to support the importance of these dietary behaviors starting early and maintained throughout childhood and their potential significance for family-based interventions targeting childhood obesity. Moreover, we have identified several associations between socio-demographic characteristics (household income, maternal education, marital status, and pre-pregnancy BMI, and child’s race ethnicity) and dietary behaviors, highlighting the need to consider socio-economic status in the development of dietary interventions. In addition, several other lifestyle behaviors including, for example, physical activity, sleep patterns and sedentary behaviors or screen time have been associated with child’s adiposity [39–43]. Although not included in the present analysis, these lifestyle behaviors could be potential mediators of the observed associations and should be considered for future family-based interventions.

Strengths of this study include its large sample size and the longitudinal data collected prospectively over nearly ten years. Very few prior studies have investigated dietary behaviors throughout childhood including multiple time points to investigate their long-term effects through adolescence. To our knowledge, this is also the first study examining longitudinal associations of dietary behaviors and insulin resistance in adolescents. Whether these dietary behaviors remain associated with adiposity or insulin resistance and prediabetes risk during the transition from adolescence to adulthood should be investigated. Our analyses were adjusted for several maternal and child socio-demographic characteristics; however, our study sample was mainly white, with a higher family income and education level, therefore limiting the generalizability of the results. Another limitation is that we collected self-reported pre-pregnancy weight at the initial prenatal visit, and maternal BMI was a strong confounder of the relationship between child behaviors and BMI. However, among 343 women who had weight recorded in the medical record in the 3 months before their last menstrual period, the association between self-reported and clinically measured weight was linear (*r* = 0.997) [44]. Also, dietary behaviors were assessed by mothers from age 4 to 8, and by children from age 9 to 11, and we cannot exclude that maternal or child answers may differ; yet, whether one or the other is more reliable is unknown. At age 9, dietary behaviors were assessed in both maternal and child questionnaires, and accordance between answers was modest (kappa coefficients = 0.18 for eating while watching television less than once per week, 0.48 for eating breakfast daily, 0.18 for eating fast food less than once per week and 0.42 for eating dinner with family daily). In addition, insulin resistance was estimated from fasting insulin and glucose levels and not with gold-standard clamps; yet, HOMA-IR has been shown to have a high sensitivity and specificity for dynamic indices of insulin resistance measured during an oral glucose tolerance test in obese adolescents [45]. Finally, we could not assess whether these healthful dietary behaviors were associated with adiposity and body weight through reduced caloric intake or improved dietary quality, or through different mechanisms.

## Conclusions

In conclusion, the frequency of healthful dietary behaviors decreases slightly in childhood with advancing age. Healthful dietary behaviors throughout childhood are associated with lower adiposity measures as well as lower estimated insulin resistance in early adolescence, and sex-specific associations were identified. Daily breakfast eating appeared to have the largest effect and most consistent association amongst the examined behaviors in both boys and girls. Associations of fast food consumption with adiposity were mainly observed in girls, and associations of eating while watching television with adiposity and insulin resistance were seen mostly in boys. Healthier dietary behaviors, which are possibly associated with a healthier overall diet quality, could be translated into simple and applicable dietary advice for families and communities. The effectiveness of such interventions in various socio-demographic settings remains to be assessed.

## Additional file

Additional file 1: **Figure S1.** Flow-chart of participants included in the longitudinal analysis. **Figure S2.** Proportion of children included in the analysis (*N*=995) with available questionnaires each year. (DOCX 31 kb)

## Abbreviations

BI: Bio-impedance
BMI: Body mass index
DXA: Dual-energy X-ray absorptiometry
GUTS: Growing Up Today Study
HOMA-IR: Homeostatic model assessment for insulin resistance
NHANES: National Health and Nutrition Examination Survey
WC: Waist circumference
